# Effect of Occurrence of Lamin A/C (*LMNA*) Genetic Variants in a Cohort of 101 Consecutive Apparent “Lone AF” Patients: Results and Insights

**DOI:** 10.3389/fcvm.2022.823717

**Published:** 2022-04-05

**Authors:** Gabrielle D'Arezzo Pessente, Luciana Sacilotto, Zaine Oliveira Calil, Natalia Quintella Sangiorgi Olivetti, Fanny Wulkan, Théo Gremen Mimary de Oliveira, Anísio Alexandre Andrade Pedrosa, Tan Chen Wu, Denise Tessariol Hachul, Maurício Ibrahim Scanavacca, José Eduardo Krieger, Francisco Carlos da Costa Darrieux, Alexandre da Costa Pereira

**Affiliations:** ^1^Laboratory of Genetics and Molecular Cardiology (LGMC) - Heart Institute (Institute Coração, University of São Paulo Medical School, São Paulo, Brazil; ^2^Arrhythmia Unit - Heart Institute (Institute Coração), University of São Paulo Medical School, São Paulo, Brazil

**Keywords:** laminopathy, *LMNA*, lone AF, atrial fibrillation, genetics

## Abstract

**Objective:**

Mutations in the **Lamin A/C** *(LMNA*) gene are commonly associated with cardiac manifestations, such as dilated cardiomyopathy (DCM) and conduction system disease. However, the overall spectrum and penetrance of rare *LMNA* variants are unknown. The present study described the presence of *LMNA* **variants** in patients with “lone atrial fibrillation (AF)” as their sole clinical presentation.

**Methods:**

One-hundred and one consecutive patients with “lone AF” criteria were initially screened by genetic testing. Genetic variants were classified according to the American College of Genetic and Genomic criteria. All subjects were evaluated through clinical and familial history, ECG, 24-h Holter monitoring, echocardiogram, cardiac magnetic resonance, treatment response, and the present relatives of *LMNA* carriers. In addition, whole-exome data from 49,960 UK Biobank (UKB) participants were analyzed to describe the overall penetrance of rare *LMNA* missense and loss of function (LOF) variants.

**Results:**

Three missense variants in *LMNA* were identified in probands with AF as their first and unique clinical manifestation. Other five first-degree relatives, after the screening, also presented *LMNA* gene variants. Among 49,960 analyzed UKB participants, 331 carried rare *LMNA* missense or LOF variant. Participants who carried a rare *LMNA* variant were significantly associated with higher odds of arrhythmic events and of an abnormal ECG in the per-protocol ECG exam (*p* = 0.03 and *p* = 0.05, respectively).

**Conclusion:**

Although a rare occurrence, our findings emphasize the possibility of an initial presentation of apparently “lone AF” in *LMNA* gene variant carriers.

## Highlights

- To demonstrate the importance of thorough clinical follow-up in patients with clinical presentation initially described as “lone atrial fibrillation (AF),” which may represent hidden forms of structural heart disease.
- To reinforce that some laminopathies can have an initial presentation as a clinical condition simulating “lone AF” in the absence of apparent demonstrable structural heart disease.
- Family screening and targeted anamnesis can guide and assist in the future diagnosis of initially isolated forms of AF.
- Genetic testing with a wide panel covering ionic and cardiomyopathies are essential for the clinical follow-up and improvement of the etiological diagnosis of the “lone AF” case.

## Introduction

Atrial Fibrillation (AF) is the most common sustained cardiac arrhythmia with an estimated prevalence of 1–2% in the general population. Although being more frequent in the elderly, AF can also occur at a younger age without known etiological risk factors identified by clinical history and/or complementary exams, a condition once called “lone AF.” This term is now discouraged by recently published AF Guidelines (1), once it is a complex disease and its mechanisms are still not fully understood. Many of these young individuals present a family history of early-onset AF and sudden death, which leads to the hypothesis that genetic factors may play an important role, at least in a subset of cases (2).

Currently, three patterns related to AF have been observed: (1) familial AF, as a monogenic disease with Mendelian inheritance; (2) familial AF in the context of another inherited cardiomyopathy or another inherited arrhythmic syndrome (3); and (3) non-familial AF associated with other than genetic predisposing conditions, such as epigenetic factors (4, 5). To date, more than 30 genes have been associated with AF (4, 6), most of those encoding ionic channels are calcium handling proteins, fibrosis, conduction, and inflammation-associated genes and possible atrial cardiomyopathy (7–11). Despite the increasing awareness in the genetics aspects of AF, a well-established curation for AF is still lacking in the molecular diagnosis arena (11).

Genes encoding structural components of the myocyte architecture (sarcomere, cytoskeleton, and nucleus) have been implicated in the genesis of AF (12). Variants in these genes may be a possible explanation to primary atrial cardiomyopathy as a result of various effects on atrium size, contractile function, cell-cell connections, and conduction velocity (13). In addition to mutation-induced primary structural defects, secondary structural remodeling of the atria in patients with chronic AF contributes to arrhythmia maintenance (14).

Mutations in the **Lamin A/C** (*LMNA*) gene present pleiotropic effects and may cause a spectrum of distinct disorders, such as striatal muscle diseases, peripheral neuropathy, partial lipodystrophy syndromes, and dilated cardiomyopathy (DCM) associated with conduction system disease (15). Albeit, the downstream pathogenic molecular mechanisms for laminopathies are poorly understood, the classical cardiac manifestations include atrioventricular (AV) block, supraventricular arrhythmias (atrial flutter, AF), sick sinus syndrome, or ventricular arrhythmias, which usually occurs after chamber dilatation (16). Laminopathies may underlie some cases of “lone AF” not associated with DCM or muscular dystrophy, although the prevalence of this phenomenon in the “lone AF” population is unknown (17).

The aim of the present study was to describe the prevalence of ***LMNA*** **variants** in a cohort of individuals with “lone AF” at baseline. In addition, we also describe the broad-sense penetrance of rare missense and loss of function (LOF) of *LMNA* variants in a cohort of individuals from the general population.

## Methods

### Lone AF Cohort

The lone AF cohort was composed by 101 subjects recruited from the Heart Institute, University of São Paulo Medical School (InCor HCFMUSP)—Brazil. Research protocols were approved by the ethics committee, after obtaining informed written consent (CAPPesq 1.664.726). Inclusion criteria were documented AF in subjects <60 years of age without known risk factors, such as hypertension, thyroid disorders, diabetes mellitus, morbid obesity [body mass index (BMI) of 40 or more], or structural heart disease (excluded by imaging exams and/or exercise stress testing).

High-throughput next-generation sequencing was performed in all probands, by the Miseq^®^ Illumina platform (Illumina, Inc., San Diego, CA, US), using a custom capturing reagent for channelopathies and cardiomyopathy genes that covered all coding exons of the *LMNA* gene. Sanger sequencing was performed for genetic screening in family members or when this information could be useful in variants classification. Primer sequences are listed in Supplementary Table 2.

Variants were classified according to the American College of Genetic and Genomic Society (ACMG) (18) criteria. Population frequency data for variants were obtained from online databases, such as the Genome Aggregation Database (GnomAD) and the Online Brazilian Archive of Mutations (ABraOM). Functional effects of missense variants were predicted by *in silico* tools [sorting intolerant from tolerant (SIFT), Polymorphism Phenotyping v2 (Polyphen-2), Mutation Assessor, and Protein Variation Effect Analyzer (PROVEAN)]. Co-segregation in multiple affected family members was considered as supporting evidence, according to ACMG criteria.

### UK Biobank (UKB) Whole-Exome Sequencing (WES) Sample

Whole-Exome Sequencing was performed by Regeneron Genetics Center. For the current analysis, data from 49,956 individual exomes of the first UKB WES release were used. In brief, read sequences were aligned to the reference human genome (version GRCH38) using Burrows-Wheeler Alignment-Maximal Exact Match (BWA-MEM). Then duplicate reads were marked using Picard tools and participant level genomic variant call files (gVCF) were generated, using the WeCall variant caller with ≥2 alternative reads to call a variant as described. Of the 49,996 subjects that were successfully sequenced, 40 samples flagged by Regeneron Genetics for QC issues were excluded from the analysis. After data access approval (UK Biobank Project 14654), WES data were downloaded to a local computer environment, annotated, and filtered for an minor allele frequency (MAF) <0.001. Variants were further filtered to contain only missense and predicted LOF variants. Finally, all variants were filtered for the *LMNA* gene and cross-referenced with the ClinVar database as of July 21, 2020.

We have collapsed all clinical phenotypes available from the baseline interview with those derived from NHS linkage. In the current analysis, we have defined the presence of any cardiomyopathy, the presence of AF, and the presence of any cardiac sign and symptoms excluding those associated with ischemic heart disease (detailed information on derivation strategy available as Supplementary Material).

### ECG

The electrocardiographic recordings (ECG and Holter) of the subjects included in the study were analyzed by two independent evaluators (LS/NO). In case of divergences, a third evaluator was required (FD). The analysis included conduction system disease, frequency in sinus rhythm, measurements of PR and QRS intervals, and corrected QT calculation and signs of conduction system disease (e.g., persistent bradycardia, advanced AV node block).

### Statistical Analysis

Due to the characteristics of this study (case series), data were descriptive by nature. Categorical variables were presented as percentages, and continuous variables were presented as mean ± SD. All analyses were carried out using IBM-SPSS statistical package (20.0 version).

## Results

**Figure 1:**
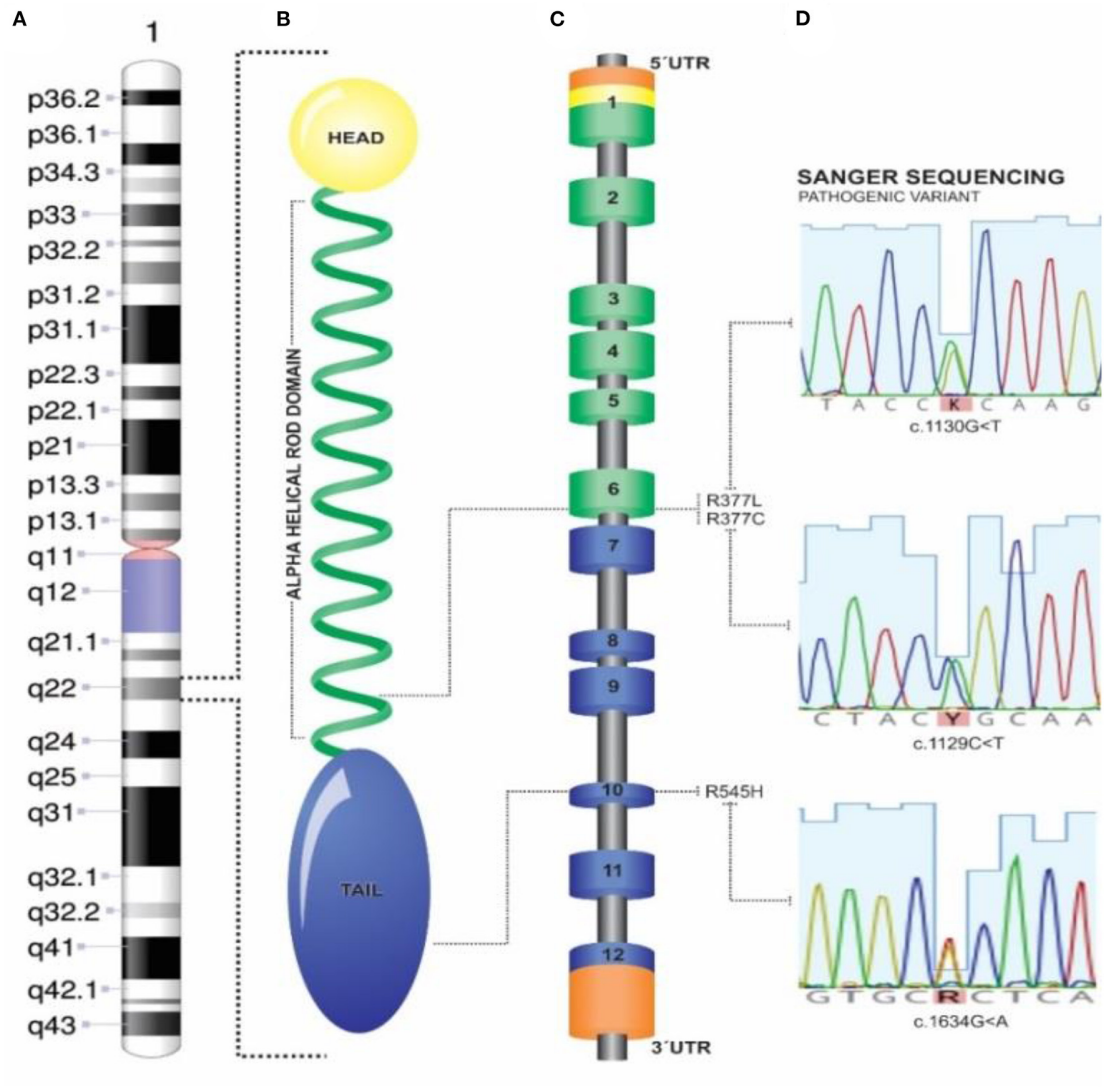
Lamin structure and genetic findings. **(A)** Chromosome 1 and 1q22 *LMNA* locus; **(B)** Head-rod-tail domain *LMNA* structure; **(C)** Schematic *LMNA* gene and 5′-3′ exome. **(D)** Sanger sequences and exon position of variants found.

**Figure 2:**
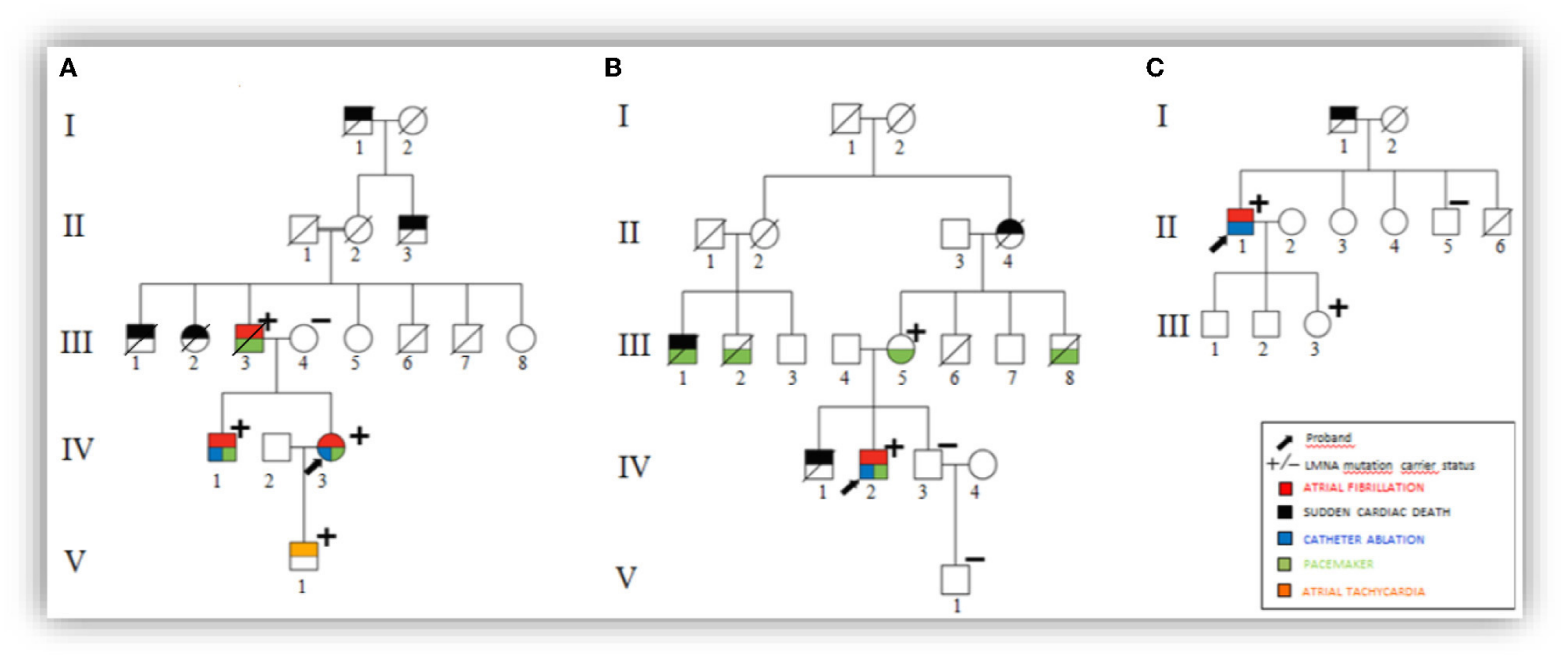
Pedigrees with variant segregation data. **(A)** Pedigree from Family A. **(B)** Pedigree from Family B. **(C)** Pedigree from Family C.

Among 101 probands with “lone AF” patients, three probands were carriers of different, heterozygous, non-synonymous variants in *LMNA* (Figure 1 and Table 1). Family screening found additional 5 *LMNA* genotype-positive family members. A total of 8 individuals were investigated and their detailed clinical evaluation is described below (Figure 2 and Table 2). There was no evidence of skeletal muscle involvement in any evaluated patients. The mean follow-up period was 10 years.

**Table 1:** Detailed information of each genetic variant in the *LMNA* gene.

**Genetic variant**	**Gnomad**	**Functional domain**	***In silico***	**Clingen**	**Cosegregation (N) ^**27236918**^**	**ACMG Conclusion**
p.Arg377Cys (Family B)	Zero	Rod domain (Exon 6)	Pathogenic effects	Dilated cardiomyopathy probands ^PMID:29095976^ (19)	¼ (no evidence)	Pathogenic
p.Arg377Leu (Family A)	Zero	Rod domain (Exon 6)	Pathogenic effects	DCM and muscular dystrophy ^PMID:12032588^ (20)	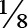 (supporting)	Pathogenic
p.Arg545His (Family C)	Low frequency (0.023%)	Exon 10	Conflicting^*^	Inflammatory myopathy ^PMID:29791652^ (21)	½ (no evidence)	VUS

* *(SIFT, “Tolerated”; Provean, “Neutral” PolyPhen-2, “Deleterious”, Mutation assessor “Medium”—for functional impact). VUS, variant of uncertain significance*.

**Table 2:** Summary of clinical findings in 8 lamin A/C genotype-positive subjects.

**Family**	**Subject**	**Gender**	**Family variant**	**Age of AF onset**	**AF type**	**Number RFCA**	**Conduction disease**	**Pacemaker**	**LVEF (%)**	**Age of DCM onset**	**Follow-up (years)**
Family A p. Arg377Leu	**IV:3**	**F**	**c.1130G>T**	**16**	**Permanent**	**4**	**No**	**36 yo**	**65**	**40**	**27**
	V:1	M	c.1130G>T	20	Paroxysmal AT	–	No	no	69	^*^	2
	IV:1	M	c.1130G>T	21	Permanent	3	No	29 yo	57	^*^	16
	III:3	M	c.1130G>T	51	Paroxysmal	–	Yes	51 yo	45	62	12
Family B p.Arg377Cys	**IV:2**	**M**	**c.1129C>T**	**35**	**Paroxysmal**	**2**	**Yes**	**38 yo**	**60**	^*^	**8**
	III:5	F	c.1129C>T	72	No AF	–	Yes	72 yo	46	74	2
Family C p.Arg545His	**II:1**	**M**	**c.1634 G>A**	**27**	**Paroxysmal**	**1**	**No**	**no**	**75**	^*^	**19**
	III:3	F	c.1634 G>A	20	No AF	–	No	No	68	^*^	1

*AT, atrial tachycardia; yo, years old; VT, ventricular tachycardia; LVEF, left ventricular ejection fraction; RFCA, radiofrequency catheter ablation. Index subjects are described in bold line.*

* *Normal cardiac evaluation until last visit*.

### Family A

A 16 years old woman (IV:3) presented with a history of palpitation due to paroxysmal AF. During 27 years of follow-up, antiarrhythmic drugs did not control symptoms or AF burden, and this patient was submitted to three radiofrequency catheter ablation (RFCA) procedures, when the patient was 18, 34, and 35 years old. The electrophysiological study revealed borderline His-ventricle interval (HV = 51 ms). Due to AF recurrences and refractoriness to conventional treatment (such as RFCA attempts), this patient was submitted to AV node ablation and pacemaker (PM) implantation. Genetic testing revealed a heterozygous pathogenic *LMNA* variant p.Arg377Leu. After a couple of asymptomatic years, the patient presented palpitation recurrences and dyspnea related to frequent premature ventricular contractions (PVCs) and short non-sustained ventricular tachycardia (NSVT; confirmed by 24-h Holter monitoring). Albeit, no structural abnormalities were found in the current echocardiogram and cardiac MRI, N-terminal pro B-type natriuretic peptide (NT-pro BNP) levels were reached 3.188 pg/ml.

Over time, other cardiac phenotypes were remarkable in family members, all carriers of the same variant (Figure 2A). At 51 years old, her father (III:3) was submitted to a dual-chamber PM implant due to symptomatic bradycardia and prolonged HV interval (>100 ms). PM registry detected subclinical paroxysmal AF. Despite the absence of risk factors for cardioembolism (CHA_2_DS_2_VASc score = zero), this patient presented a stroke after 8 years of follow-up. At 62 years old, the patient developed dyspnea and peripheral edema. Echocardiogram, which was normal at the baseline, demonstrated severe enlargement of the atria, mild left ventricular dysfunction (left ventricular ejection fraction (LVEF) = 45%), with diffuse hypokinesia and pulmonary hypertension (PASP-pulmonary artery systolic pressure = 50 mmHg). The patient also developed a high frequency of polymorphic premature ventricular contractions (PVC; 12%) with repetitive NSVT (maximum of 8 beats) and had a sudden cardiac death (SCD) at 68 years old.

Her brother (IV:1) had symptomatic AF since he was 21 years old, also submitted to three RFCA procedures, requiring AV node ablation and PM implant at age of 29, because of treatment failure. He remains asymptomatic for the last 8 years, with subsequent normal echocardiogram and cardiac MRI.

Her only child (V:1) developed palpitations at 22-year-old and sustained atrial tachycardia was documented in exercise stress testing. Resting ECG showed premature supraventricular contractions, left anterior fascicular block, and incomplete right bundle branch block. He had a normal physical examination, and the echocardiogram had no evidence of structural heart disease.

Subjects I:1, II:3, III:1, and III:2 died suddenly at the ages of <55, 60, 73, and 8, respectively. The underlying mechanism of deaths could not be retrieved.

### Family B

The proband of this family is a 35-year-old male (IV:2), presenting paroxysmal AF, controlled with propafenone 150 mg twice a day (BID), without apparent structural heart disease. After 2 years, he developed a third-degree AV block requiring dual-chamber PM implantation. He presented AF recurrences while on the antiarrhythmic drug, requiring two RFCA procedures at 41 and 42 years old. This patient remains currently asymptomatic, with some bursts of asymptomatic atrial tachycardia at 24-h Holter monitoring.

His family history was remarkable. His brother (IV:1) died suddenly at 16 years old. In addition, three family members (III:1, III:2, and III:8) had an implanted PM between the ages of 50 and 70. His mother (III:5), 74 years old, had a PM implanted at 72 years old, due to symptomatic bradycardia. An echocardiogram showed mild ventricular dysfunction (LVEF 46%), augmented linear dimension of the left atrial, and pulmonary hypertension (PASP = 47 mmHg). She had no documented AF.

Both proband and her mother harbored the same *LMNA* variant p.Arg377Cys. His second brother (IV:3) and the correspondent nephew (V:1) were asymptomatic and without *LMNA* genetic variant findings (Figure 2B—pedigree).

### Family C

A 43 years old man (II:1) underwent AF RFCA after several episodes of symptomatic AF, since he was 27, without recurrences during 3 years of follow-up. Baseline ECG showed sinus rhythm and low QRS voltage only in leads I and aVL. Echocardiogram measurements were within the normal ranges and systolic function. Genetic testing revealed a p.Arg545His variant in heterozygosis.

His father (I:1) died suddenly at age of 67 of an unknown cause. His daughter (III:3), 21 years old, also a carrier of the p.Arg545His variant, had a complaint of palpitations, without documented ECG symptoms related. At the evaluation, she was in sinus rhythm and no structural heart abnormalities were observed at imaging exams. She was considered as a silent carrier. Proband's brother (II:5) was asymptomatic and he did not carry the same variant. There was no further family history of AF, DCM, or PM implantation (Figure 2C—pedigree).

### Prevalence and Penetrance of Rare *LMNA* Missense and LOF Variants According to the UKB Database

Among the 49,956 UKB participants with WES data available for this analysis, there were 308 carriers of a rare *LMNA* missense (300 carriers) or LOF (8 participants) variant. From these, 6 individuals carried variants previously described in ClinVar as pathogenic (P), and 162 are carriers of variants listed in ClinVar as a variant of uncertain significance (VUS; Supplementary Material).

The six identified pathogenic variants were p.Arg298Cys, p.Arg482Trp, p.Arg435Cys (present in two individuals), p.Arg527Cys, and p.Arg527His. The p.Arg298Cys variant is a founder mutation in North African countries and has been reported to segregate with autosomal recessive Charcot-Marie-Tooth type 2 in affected families. Nonetheless, its role in autosomal dominant cardiomyopathy is less well-established. The p.Arg482Trp variant has been reported in at least 12 European individuals with familial partial lipodystrophy, segregated with disease in at least 7 affected relatives from 2 families. However, here again, its role in autosomal dominant cardiomyopathy has not been well-established. The carrier of variant p.Arg482Trp had AF in the setting of ischemic coronary artery disease, ischemic cardiomyopathy, and hypertension. The carrier of variant p.Arg298Cys had no previous history of any cardiovascular condition at the age of 63, but was noticed to have a slightly reduced left ventricle ejection fraction at a per-protocol cardiac MRI examination (48%). The p.Arg435Cys variant was present in two individuals, i.e., 43 and 61 years old women with no cardiovascular alterations in their medical records. The carrier of the p.Arg527Cys variant is a 63-year-old hypertensive male also with no signs or symptoms of cardiovascular disease. Finally, the carrier of p.Arg527His is a 49-year-old hypertensive female participant with no history of cardiovascular disease. Altogether, from the 6 carriers of a pathogenic *LMNA* variant, only 2 presented signs of electrocardiographic abnormalities and/or cardiac arrhythmia.

Among the individuals carrying a VUS, from which pathogenicity is not well-established, we observed an enrichment of cardiovascular signs and symptoms when compared to the overall UKB cohort. Of the 162 participants with a VUS in *LMNA*, 16 had an international code disease (ICD)10 for arrhythmia (Supplementary Table 3). Indeed, carrying a pathogenic or VUS variant in *LMNA* was significantly associated with higher odds of an arrhythmia diagnosis in UKB participants electronic health records (HER; odds ratio [OR]: 1.73, *p* = 0.03).

From the 308 UKB participants with released WES data as for July 2020, 75 were submitted to a per-protocol ECG evaluation. Carrying a rare missense or LOF variant in *LMNA* was associated with a significantly higher odds of having an abnormal ECG in age- and sex-adjusted logistic regression model using data on 13,592 ECG exams from the UKB WES cohort (OR = 1.60, *p* = 0.03). Among carrier individuals, only 2 had established non-ischemic cardiomyopathy, suggesting that ECG abnormalities are independent markers or precede structural heart abnormalities incidence in this cohort.

## Discussion

We discuss the phenotypic aspects of 3 probands from a cohort of patients with “lone AF,” in addition to 5 family members, without DCM at initial presentation, harboring ***LMNA*** **gene variants**. The *LMNA* gene is associated with several different phenotypes, such as DCM with or without conduction system disease and AF. The NIH-funded Clinical Genome Resource (ClinGen), a framework to define and evaluate the clinical validity of gene-disease pairs across a variety of Mendelian disorders, has established *LMNA* gene as definitive for DCM. ClinGen has not curated genes for AF, in spite of the cumulative data in this field in recent years (22).

The AF occurrence in *LMNA* patients is usually observed in combination with DCM. Several studies have associated the presence of AF with *LMNA* carrier status (23–27), although few have shown AF as the initial and cardinal symptom (17, 28). In a Norwegian cohort of 93 *LMNA* carriers, cardiac penetrance was high in young asymptomatic *LMNA* genotype-positive family members with frequent AV block and ventricular tachycardia, highlighting the importance of early family screening. Early-onset of AF was not reported (19). The prevalence of an *LMNA* mutation was 6.2% in this cohort of familial DCM and, in our “lone AF” cohort, the prevalence was 3%.

Glocklhofer et al. (29), in a large *LMNA* family, described patients with DCM and a pronounced arrhythmogenic phenotype with several SCD and implantable cardioverter defibrillator (ICD) with appropriated shocks. Regarding AF, they noticed paroxysmal AF at a mean age of 42 years rapidly progressing to permanent AF, within 5 years. In our series, instead, AF was the initial presentation in 6 out of 8 *LMNA* carriers (75%), with challenging rhythm control treatment as well, as shown by several AF catheter ablation procedures in 3 patients, two of them were progressing to AV node ablation due to failure in rhythm control.

Although atrial cardiomyopathy has been suggested to play an important role in the development of AF, definite conclusions about these issues are still ongoing. Ahlberg et al. (30), in an elegant study using machine-learning algorithms, segmented and annotated the left atrium (LA) in cardiac magnetic resonance data from 35,658 individuals. They identified 18 novel genetic loci associated with LA volume and function, with a high genetic correlation between LA volume and function and stroke, suggesting that genetic risk of AF can influence LA structure prior to diagnosis of AF.

Molecular predisposition is an interesting frontier in the past so-called “lone AF,” indeed a silent form of atrial cardiomyopathy. Albergh et al. (31) also observed compromised assembly of the sarcomere in both atria and ventricle, longer PR interval, and heterozygous adult zebrafish with a higher degree of fibrosis in the atria, indicating that titin-truncating variants are also important risk factors for AF.

One of the interesting issues of our study was the early and initial development of AF and, up to this point, two family members had classical cardiomyopathy, over 60 years old, illustrating the age-dependent penetrance in the *LMNA* gene mutations. One patient developed DCM after 11 years of AF onset and he developed SCD soon after a decrease in ejection fraction within mild impairment (LVEF = 45%).Van Berlo et al. (32) demonstrated a 46% risk for SCD in a meta-analysis that includes 299 carriers of pathogenic *LMNA* gene variants; this risk was considered the same when the patients had a cardiac or neuromuscular phenotype or PM implantation. Van Rijsingen et al. (33), in a multicenter registry of 269 *LMNA* variant carriers, demonstrated after multivariable analysis that NSVT during ambulatory ECG monitoring, LVEF <45% at first evaluation, male sex, and LOF variants (insertion-deletion/truncating or mutations affecting splicing) were independent risk factors for malignant ventricular arrhythmias. Hasselberg et al. (34) demonstrated that a prolonged PR interval on ECG, reduced myocardial function, and septal fibrosis were independent predictors of SCD, changes which were not observed in our patients, due to lack of power (low rate of SCD in probands). In our registry, only one patient had died after diagnosis and his marker of risk was a recently reduced ejection fraction. A recent guideline reinforces an earlier ICD indication in this subgroup of patients (35). The second family member is probably developing incipient cardiomyopathy, as demonstrated by BNP level and increased PVC burden, controlled with bisoprolol therapy. She has been evaluated in a close follow-up period to determine the best moment to proceed to ICD implantation. The other patient with DCM had PM implantation as the first signal of laminopathy. Only one subject is asymptomatic, although too young to preclude cardiac phenotype.

The three different variants found in our study were considered dominant missense ones, with families A and B at the same 377 residues (p.Arg377Leu and p.Arg377Cys) and family C at p.Arg545His. Several patients in ClinVar records harbor *LMNA* variant at residue 377, totalizing 34 indexed publications. The 545 residue, instead, appears in 18 ClinVar records, with conflicting interpretations of pathogenicity. All are focused on DCM and complications of muscular dystrophy. However, in our point of view, p.Arg545His variant has not enough evidence to be classified as likely pathogenic, despite hotspot location and *in silico* prediction of deleterious effect. The p.Arg545His variant has a relatively high MAF with 48/193996 alleles in the controls database (https://gnomad.broadinstitute.org/variant/1-156107470-G-A) and has previously been identified in two sisters with autosomal recessive lipodystrophy syndromes, without a description of cardiovascular evaluation (36). As our study was focused on “lone AF” patients, the phenotypic characterization is challenging itself and its true prevalence. Asymptomatic carriers are poorly documented in the literature, probably due to the high penetrance of the disease^1^.

Our research was initially designed to study rare genetic variants for “lone AF” patients and the findings related to laminopathies occurred in a haphazard manner. Thus, we were able to demonstrate with the follow-up of carrier families that AF may occur years before the onset of cardiomyopathy. On the other hand, we also agree with the recent concept that the term “lone AF” would not be appropriate, since association studies with other genetic alterations reinforce that the term applied to a time of “macroscopic” concept and without considering the late development of phenotypic profiles, as it can happen in patients with laminopathies.

We have also been able to describe the overall prevalence and association structure of rare *LMNA* variants using ~50,000 WES samples from the UKB study. In this sample, rare missense or LOF variants in the gene were observed in 331 participants, a prevalence of about 1:150 individuals from the general population. Although not a proper sample to study “lone AF” because of the somehow older mean age of the cohort (the mean age of carriers in our analysis was 58 years), UKB can provide a source to study penetrance estimates for these rare variants. Even though we only identified 2 carriers of previously described to be pathogenic variants in the UKB sample, both of these individuals were phenotypically affected, suggesting a high penetrance of pathogenic variants for this gene. Interestingly, even harboring variants with not so well-established pathogenicity were also associated with increased odds of arrhythmia and/or electrocardiographic alterations. It is important to note, however, that pathogenicity for most of the observed rare variants is still unknown and the sole presence of a rare missense or LOF variant in this gene does not carry, without further information on the variant, much information to be useful at an individual level basis.

Dilated cardiomyopathy is the hallmark of classical laminopathies, as demonstrated in recent studies of Captur et al. (37), who reviewed Lamin A/C heart disease and showed that DCM occurred in 10% of *LMNA* carriers, up to 5% in patients with sporadic idiopathic DCM, 5–10% with idiopathic familial DCM, and 33% with familial DCM and cardiac conduction system disease.

Boriani et al. (38) suggested the pathogenetic pathways, which start from activation of Erk 1/2 signaling leading to a profibrotic process, which encompasses transforming growth (TGF) beta 2-dependent activation of connective tissue growth factor, with the consequent progression of cardiomyopathy. Kumar et al. (39) also demonstrated that preferential involvement of atriums was an even a rare situation.

There was no such evidence in our families of neuromuscular involvement, as creatine phosphokinase (CPK) levels were all within normal range, which is another important phenotype that is usually present in classic laminopathy diseases (40).

In *LMNA*-KO animal experimental models, it has been demonstrated a premature presentation of the various phenotypes of laminopathies, such as muscular dystrophy and cardiomyopathy, besides pathological changes in other tissues, such as liver, lung, and kidney (41). Similarly, these pathological changes have been observed in human studies. Nonetheless, our understanding of the genetic basis of AF in *LMNA* carriers is limited so far.

Our study has some limitations. It is an observational follow-up study initially designed based in a consecutive cohort of patients with apparently “lone AF.” Although controversial, to our understanding, we only included patients without any apparent structural heart disease. A longer follow-up time would be needed to assess the true cardiomyopathy penetrance of *LMNA* genotype-positive patients and the true clinical heterogeneity of the disease in patients presenting with “lone AF” as the sole clinical manifestation of diseases. Another limitation of our study is the lack of confirmation in experimental models of p.Arg545His variant here defined as VUS. Copy number variants (CNV) was not analyzed for LMNA due to limited grant and lack of proper supplies. UKB sample, as previously mentioned, is not the most appropriate sample to study genetic determinants of lone AF. However, the unbiased ascertainment and, more important, sequencing strategy were able to compensate for this limitation and provide an informative view of the enrichment of arrhythmia-associated phenotype in carriers of rare variants of this gene.

In conclusion, we describe AF, isolated and of early-onset, as the sole initial presentation of patients with laminopathy and later development of heart failure. This reinforces that some laminopathies can have an initial presentation as a clinical condition simulating lone AF in the absence of apparent demonstrable structural heart disease.

## Data Availability Statement

The data presented in the study are deposited in the library of the Faculty of Medicine of the University of São Paulo (HCFMUSP) repository, accession number [FM-Fac. Medicina] W4.DB8 SP.USP FM-1 P938as 2019 original.

## Ethics Statement

The studies involving human participants were reviewed and approved by Comissão de Ética para Análise de Projetos de Pesquisa - CAPPesq (CAPPesq 1.664.726) - University of São Paulo Medical School. The patients/participants provided their written informed consent to participate in this study.

## Author Contributions

GP performed the experiments, collected and analyzed the data, and wrote the paper. LS and ZC collected and analyzed the data and wrote the paper. LS, FD, NO, TW, and DH recruited the patients, collected data, and revised the manuscript. FW, TdO, and ACP performed the experiments and analyzed the data. JK, MS, FD, and ACP analyzed the data and critically revised the manuscript. All authors have contributed substantially to the conception and design of this paper.

## Funding

The authors declare that they have received Grants (426088/2016-6) for support of this study from the National Council for Scientific and Technological Development (CNPq).

## Conflict of Interest

The authors declare that the research was conducted in the absence of any commercial or financial relationships that could be construed as a potential conflict of interest.

## Publisher's Note

All claims expressed in this article are solely those of the authors and do not necessarily represent those of their affiliated organizations, or those of the publisher, the editors and the reviewers. Any product that may be evaluated in this article, or claim that may be made by its manufacturer, is not guaranteed or endorsed by the publisher.

## Footnotes

^1^Available online at: https://www.ncbi.nlm.nih.gov/clinvar/?term=lmna+R377.
