# Supplementary material for: Effect of Occurrence of Lamin A/C (LMNA) Genetic Variants in a Cohort of 101 Consecutive Apparent “Lone AF” Patients: Results and Insights
Source: Front Cardiovasc Med. 2022 Apr 5;9:823717. doi: 10.3389/fcvm.2022.823717 (PMC9016147; doi:10.3389/fcvm.2022.823717)
Supplement: Supplementary file 1 [file Data_Sheet_1.PDF]

SUPPLEMENTARY

Table S1 - Demographic cohort characteristics

**Characteristics**		**AF patients (n = 101)**	**98 patients**	***LMNA* patients**
**Gender,** n (%)	Male	78 (77.2)	76 (77.5)	2 (66.6)
**Atrial Fibrillation type,** n (%)	Paroxysmal	76 (75.2)	74 (75.5)	2 (66.6)
	Persistent	19 (18.9)	19 (19.3)	0
	Permanent	6 (5.9)	5 (5.1)	1 (33.3)
**Family history,** n (%)	Sudden death	37 (41.1)	34 (34.7)	3 (100)
	Early onset AF	44 (48.9)	42 (42.8)	2 (66.6)
	Pacemaker	10 (11.1)	8 (8.16)	2 (66.6)
	Heart failure	13 (14.4)	11 (11.2)	2 (66.6)
**Echocardiogram,** mean ± SD	Left atria	38 ± 5.1	38,1 ± 5.1	34.6 ± 5.5
	LVDD	49.1 ± 4.2	49.1 ± 4.3	49.6 ± 2.9
	LVSD	32.2 ± 3.9	32.2 ± 3.9	32,3 ± 3.2
	LVEF (%)	63.2 ± 5.9	63.1 ± 6.0	64.0 ± 3.6

Legend: LVDD, left ventricle diastolic diameter; **LVSD,** left ventricle systolic diameter; **LVEF,** Left ventricular ejection fraction; **SD,** standard deviation.

Table S2.Primers for Sanger's method

**Exon**	**Size**	**Forward Primer (5′→3′)**	**MT**	**Reverse Primer (5′→3′)**	**MT**
6	400bp	ATTGCAGATCCTGGAGAGAGTA	61 ºC	GGGTCTAGTCAAGGCCAGTT	61.8 ºC
10	287bp	GTAGACATGCTGTACAACCC	59.9 ºC	GGCCAGCGAGTAAAGTTCCA	64.2 ºC

Legend: MT, melting temperature; **bp,** base pairs; º**C** - celsius degree.

Chr	Position	Ref	Alteration	Gene Name	CDS change	AA change	Effect	Clinical Significance	Arrhythmia
chr1	156114938	G	A	*LMNA*	c.20G>A	p.Arg7Gln	missense_variant	Uncertain	NO
chr1	156114959	C	T	*LMNA*	c.41C>T	p.Ala14Val	missense_variant	NA	NO
chr1	156114965	C	T	*LMNA*	c.47C>T	p.Ala16Val	missense_variant	NA	NO
chr1	156114998	C	G	*LMNA*	c.80C>G	p.Thr27Ser	missense_variant	Uncertain	NO
chr1	156115036	G	C	*LMNA*	c.118G>C	p.Asp40His	missense_variant	NA	NO
chr1	156115174	G	A	*LMNA*	c.256G>A	p.Gly86Arg	missense_variant	NA	NO
chr1	156115208	A	C	*LMNA*	c.290A>C	p.Lys97Thr	missense_variant	Uncertain	NO
chr1	156115268	A	G	*LMNA*	c.350A>G	p.Lys117Arg	missense_variant	Uncertain	NO
chr1	156115268	A	G	*LMNA*	c.350A>G	p.Lys117Arg	missense_variant	Uncertain	NO
chr1	156126827	A	G	*LMNA*	c.25A>G	p.Asn9Asp	missense_variant	NA	NO
chr1	156126840	C	T	*LMNA*	c.38C>T	p.Ala13Val	missense_variant	NA	NO
chr1	156126879	C	A	*LMNA*	c.77C>A	p.Ala26Asp	missense_variant	NA	NO
chr1	156126893	C	T	*LMNA*	c.91C>T	p.Arg31Trp	missense_variant	NA	NO
chr1	156126893	C	T	*LMNA*	c.91C>T	p.Arg31Trp	missense_variant	NA	NO
chr1	156126896	G	A	*LMNA*	c.94G>A	p.Ala32Thr	missense_variant	NA	NO
chr1	156126910	T	TC	*LMNA*	c.112dupC	p.Leu38fs	frameshift & splice site variant	NA	NO
chr1	156126914	C	T	*LMNA*	c.112C>T	p.Leu38Phe	missense & splice site variant	NA	NO
chr1	156126919	A	G	*LMNA*	c.117A>G	p.Ter39Trp	stop_lost	NA	NO
chr1	156126919	A	G	*LMNA*	c.117A>G	p.Ter39Trp	stop_lost	NA	NO
chr1	156130699	G	A	*LMNA*	c.439G>A	p.Ala147Thr	missense_variant	NA	NO
chr1	156130727	G	A	*LMNA*	c.467G>A	p.Arg156His	missense_variant	NA	NO
chr1	156130727	G	A	*LMNA*	c.467G>A	p.Arg156His	missense_variant	NA	NO
chr1	156130727	G	A	*LMNA*	c.467G>A	p.Arg156His	missense_variant	NA	AF/FLU
chr1	156130730	C	T	*LMNA*	c.470C>T	p.Thr157Met	missense_variant	NA	NO
chr1	156130756	C	T	*LMNA*	c.496C>T	p.Arg166Trp	missense_variant	Uncertain	AF and FLU
chr1	156130756	C	T	*LMNA*	c.496C>T	p.Arg166Trp	missense_variant	Uncertain	Cardiac arrest
chr1	156130757	G	A	*LMNA*	c.497G>A	p.Arg166Gln	missense_variant	Uncertain	AF and FLU
chr1	156130757	G	A	*LMNA*	c.497G>A	p.Arg166Gln	missense_variant	Uncertain	AF and FLU
chr1	156130757	G	A	*LMNA*	c.497G>A	p.Arg166Gln	missense_variant	Uncertain	NO
chr1	156130757	G	A	*LMNA*	c.497G>A	p.Arg166Gln	missense_variant	Uncertain	NO
chr1	156130757	G	A	*LMNA*	c.497G>A	p.Arg166Gln	missense_variant	Uncertain	Other arrh
chr1	156130757	G	A	*LMNA*	c.497G>A	p.Arg166Gln	missense_variant	Uncertain	NO
chr1	156130757	G	A	*LMNA*	c.497G>A	p.Arg166Gln	missense_variant	Uncertain	NO
chr1	156130757	G	A	*LMNA*	c.497G>A	p.Arg166Gln	missense_variant	Uncertain	NO
chr1	156134444	T	G	*LMNA*	c.555T>G	p.Asp185Glu	missense_variant	NA	NO
chr1	156134444	T	G	*LMNA*	c.555T>G	p.Asp185Glu	missense_variant	NA	NO
chr1	156134444	T	G	*LMNA*	c.555T>G	p.Asp185Glu	missense_variant	NA	NO
chr1	156134444	T	G	*LMNA*	c.555T>G	p.Asp185Glu	missense_variant	NA	NO
chr1	156134444	T	G	*LMNA*	c.555T>G	p.Asp185Glu	missense_variant	NA	NO
chr1	156134444	T	G	*LMNA*	c.555T>G	p.Asp185Glu	missense_variant	NA	NO
chr1	156134444	T	G	*LMNA*	c.555T>G	p.Asp185Glu	missense_variant	NA	NO
chr1	156134444	T	G	*LMNA*	c.555T>G	p.Asp185Glu	missense_variant	NA	NO
chr1	156134444	T	G	*LMNA*	c.555T>G	p.Asp185Glu	missense_variant	NA	NO
chr1	156134444	T	G	*LMNA*	c.555T>G	p.Asp185Glu	missense_variant	NA	NO
chr1	156134444	T	G	*LMNA*	c.555T>G	p.Asp185Glu	missense_variant	NA	NO
chr1	156134444	T	G	*LMNA*	c.555T>G	p.Asp185Glu	missense_variant	NA	NO
chr1	156134444	T	G	*LMNA*	c.555T>G	p.Asp185Glu	missense_variant	NA	NO
chr1	156134444	T	G	*LMNA*	c.555T>G	p.Asp185Glu	missense_variant	NA	NO
chr1	156134444	T	G	*LMNA*	c.555T>G	p.Asp185Glu	missense_variant	NA	NO
chr1	156134444	T	G	*LMNA*	c.555T>G	p.Asp185Glu	missense_variant	NA	NO
chr1	156134444	T	G	*LMNA*	c.555T>G	p.Asp185Glu	missense_variant	NA	NO
chr1	156134444	T	G	*LMNA*	c.555T>G	p.Asp185Glu	missense_variant	NA	NO
chr1	156134444	T	G	*LMNA*	c.555T>G	p.Asp185Glu	missense_variant	NA	AF and FLU
chr1	156134444	T	G	*LMNA*	c.555T>G	p.Asp185Glu	missense_variant	NA	NO
chr1	156134444	T	G	*LMNA*	c.555T>G	p.Asp185Glu	missense_variant	NA	NO
chr1	156134444	T	G	*LMNA*	c.555T>G	p.Asp185Glu	missense_variant	NA	NO
chr1	156134444	T	G	*LMNA*	c.555T>G	p.Asp185Glu	missense_variant	NA	NO
chr1	156134444	T	G	*LMNA*	c.555T>G	p.Asp185Glu	missense_variant	NA	NO
chr1	156134444	T	G	*LMNA*	c.555T>G	p.Asp185Glu	missense_variant	NA	NO
chr1	156134469	G	A	*LMNA*	c.580G>A	p.Glu194Lys	missense_variant	NA	AF and FLU
chr1	156134812	G	A	*LMNA*	c.647G>A	p.Arg216His	missense_variant	Uncertain	NO
chr1	156134812	G	A	*LMNA*	c.647G>A	p.Arg216His	missense_variant	Uncertain	NO
chr1	156134812	G	A	*LMNA*	c.647G>A	p.Arg216His	missense_variant	Uncertain	NO
chr1	156134812	G	A	*LMNA*	c.647G>A	p.Arg216His	missense_variant	Uncertain	NO
chr1	156134812	G	A	*LMNA*	c.647G>A	p.Arg216His	missense_variant	Uncertain	NO
chr1	156134812	G	A	*LMNA*	c.647G>A	p.Arg216His	missense_variant	Uncertain	NO

chr1	156134823	C	T	*LMNA*	c.658C>T	p.Arg220Cys	missense_variant	Uncertain	NO
chr1	156134826	C	T	*LMNA*	c.661C>T	p.Arg221Cys	missense_variant	Uncertain	NO
chr1	156134851	T	C	*LMNA*	c.686T>C	p.Ile229Thr	missense_variant	Uncertain	NO
chr1	156134914	C	T	*LMNA*	c.749C>T	p.Ala250Val	missense_variant	Uncertain	NO
chr1	156134914	C	T	*LMNA*	c.749C>T	p.Ala250Val	missense_variant	Uncertain	NO
chr1	156134937	C	G	*LMNA*	c.772C>G	p.Gln258Glu	missense_variant	NA	NO
chr1	156134952	C	A	*LMNA*	c.787C>A	p.Leu263Met	missense_variant	Uncertain	NO
chr1	156135224	A	G	*LMNA*	c.848A>G	p.Asn283Ser	missense_variant	Uncertain	NO
chr1	156135224	A	G	*LMNA*	c.848A>G	p.Asn283Ser	missense_variant	Uncertain	NO
chr1	156135224	A	G	*LMNA*	c.848A>G	p.Asn283Ser	missense_variant	Uncertain	NO
chr1	156135224	A	G	*LMNA*	c.848A>G	p.Asn283Ser	missense_variant	Uncertain	NO
chr1	156135224	A	G	*LMNA*	c.848A>G	p.Asn283Ser	missense_variant	Uncertain	NO
chr1	156135224	A	G	*LMNA*	c.848A>G	p.Asn283Ser	missense_variant	Uncertain	NO
chr1	156135224	A	G	*LMNA*	c.848A>G	p.Asn283Ser	missense_variant	Uncertain	NO
chr1	156135224	A	G	*LMNA*	c.848A>G	p.Asn283Ser	missense_variant	Uncertain	NO
chr1	156135224	A	G	*LMNA*	c.848A>G	p.Asn283Ser	missense_variant	Uncertain	NO
chr1	156135262	C	T	*LMNA*	c.886C>T	p.Arg296Cys	missense_variant	Uncertain	NO
chr1	156135268	C	T	*LMNA*	c.892C>T	p.Arg298Cys	missense_variant	Pathogenic	NO
chr1	156135269	G	T	*LMNA*	c.893G>T	p.Arg298Leu	missense_variant	Uncertain	NO
chr1	156135269	G	A	*LMNA*	c.893G>A	p.Arg298His	missense_variant	NA	AF and FLU
chr1	156135904	G	A	*LMNA*	c.940G>A	p.Ala314Thr	missense_variant	NA	NO
chr1	156135910	A	C	*LMNA*	c.946A>C	p.Lys316Gln	missense_variant	NA	NO
chr1	156135910	A	C	*LMNA*	c.946A>C	p.Lys316Gln	missense_variant	NA	NO
chr1	156135910	A	C	*LMNA*	c.946A>C	p.Lys316Gln	missense_variant	NA	NO
chr1	156135910	A	C	*LMNA*	c.946A>C	p.Lys316Gln	missense_variant	NA	NO
chr1	156135910	A	C	*LMNA*	c.946A>C	p.Lys316Gln	missense_variant	NA	NO
chr1	156135910	A	C	*LMNA*	c.946A>C	p.Lys316Gln	missense_variant	NA	NO
chr1	156135910	A	C	*LMNA*	c.946A>C	p.Lys316Gln	missense_variant	NA	NO
chr1	156135910	A	C	*LMNA*	c.946A>C	p.Lys316Gln	missense_variant	NA	NO
chr1	156135910	A	C	*LMNA*	c.946A>C	p.Lys316Gln	missense_variant	NA	NO
chr1	156135910	A	C	*LMNA*	c.946A>C	p.Lys316Gln	missense_variant	NA	NO
chr1	156135910	A	C	*LMNA*	c.946A>C	p.Lys316Gln	missense_variant	NA	NO
chr1	156135910	A	C	*LMNA*	c.946A>C	p.Lys316Gln	missense_variant	NA	NO
chr1	156135910	A	C	*LMNA*	c.946A>C	p.Lys316Gln	missense_variant	NA	NO
chr1	156135910	A	C	*LMNA*	c.946A>C	p.Lys316Gln	missense_variant	NA	NO
chr1	156135910	A	C	*LMNA*	c.946A>C	p.Lys316Gln	missense_variant	NA	NO
chr1	156135910	A	C	*LMNA*	c.946A>C	p.Lys316Gln	missense_variant	NA	NO
chr1	156135910	A	C	*LMNA*	c.946A>C	p.Lys316Gln	missense_variant	NA	NO
chr1	156135910	A	C	*LMNA*	c.946A>C	p.Lys316Gln	missense_variant	NA	NO
chr1	156135910	A	C	*LMNA*	c.946A>C	p.Lys316Gln	missense_variant	NA	NO
chr1	156135910	A	C	*LMNA*	c.946A>C	p.Lys316Gln	missense_variant	NA	NO
chr1	156135910	A	C	*LMNA*	c.946A>C	p.Lys316Gln	missense_variant	NA	NO
chr1	156135910	A	C	*LMNA*	c.946A>C	p.Lys316Gln	missense_variant	NA	NO
chr1	156135910	A	C	*LMNA*	c.946A>C	p.Lys316Gln	missense_variant	NA	NO
chr1	156135910	A	C	*LMNA*	c.946A>C	p.Lys316Gln	missense_variant	NA	NO
chr1	156135910	A	C	*LMNA*	c.946A>C	p.Lys316Gln	missense_variant	NA	NO
chr1	156135910	A	C	*LMNA*	c.946A>C	p.Lys316Gln	missense_variant	NA	NO
chr1	156135910	A	C	*LMNA*	c.946A>C	p.Lys316Gln	missense_variant	NA	NO
chr1	156135910	A	C	*LMNA*	c.946A>C	p.Lys316Gln	missense_variant	NA	Other CD
chr1	156135910	A	C	*LMNA*	c.946A>C	p.Lys316Gln	missense_variant	NA	NO
chr1	156135910	A	C	*LMNA*	c.946A>C	p.Lys316Gln	missense_variant	NA	NO
chr1	156135910	A	C	*LMNA*	c.946A>C	p.Lys316Gln	missense_variant	NA	NO
chr1	156135910	A	C	*LMNA*	c.946A>C	p.Lys316Gln	missense_variant	NA	NO
chr1	156135910	A	C	*LMNA*	c.946A>C	p.Lys316Gln	missense_variant	NA	NO
chr1	156135916	G	A	*LMNA*	c.952G>A	p.Ala318Thr	missense_variant	Likely benign	NO
chr1	156135916	G	A	*LMNA*	c.952G>A	p.Ala318Thr	missense_variant	Likely benign	NO
chr1	156135916	G	A	*LMNA*	c.952G>A	p.Ala318Thr	missense_variant	Likely benign	NO
chr1	156135916	G	A	*LMNA*	c.952G>A	p.Ala318Thr	missense_variant	Likely benign	NO
chr1	156135916	G	A	*LMNA*	c.952G>A	p.Ala318Thr	missense_variant	Likely benign	NO
chr1	156135916	G	A	*LMNA*	c.952G>A	p.Ala318Thr	missense_variant	Likely benign	NO
chr1	156135916	G	A	*LMNA*	c.952G>A	p.Ala318Thr	missense_variant	Likely benign	NO
chr1	156135916	G	A	*LMNA*	c.952G>A	p.Ala318Thr	missense_variant	Likely benign	NO
chr1	156135916	G	A	*LMNA*	c.952G>A	p.Ala318Thr	missense_variant	Likely benign	NO
chr1	156135916	G	A	*LMNA*	c.952G>A	p.Ala318Thr	missense_variant	Likely benign	NO
chr1	156135916	G	A	*LMNA*	c.952G>A	p.Ala318Thr	missense_variant	Likely benign	NO
chr1	156135917	C	T	*LMNA*	c.953C>T	p.Ala318Val	missense_variant	Uncertain	NO

chr1	156135935	A	G	*LMNA*	c.971A>G	p.Glu324Gly	missense_variant	NA	NO
chr1	156135940	T	A	*LMNA*	c.976T>A	p.Ser326Thr	missense_variant	Uncertain	NO
chr1	156135940	T	A	*LMNA*	c.976T>A	p.Ser326Thr	missense_variant	Uncertain	NO
chr1	156135940	T	A	*LMNA*	c.976T>A	p.Ser326Thr	missense_variant	Uncertain	NO
chr1	156135940	T	A	*LMNA*	c.976T>A	p.Ser326Thr	missense_variant	Uncertain	NO
chr1	156135940	T	A	*LMNA*	c.976T>A	p.Ser326Thr	missense_variant	Uncertain	NO
chr1	156135949	C	T	*LMNA*	c.985C>T	p.Arg329Cys	missense_variant	Uncertain	NO
chr1	156135949	C	G	*LMNA*	c.985C>G	p.Arg329Gly	missense_variant	Uncertain	AF and FLU
chr1	156135949	C	A	*LMNA*	c.985C>A	p.Arg329Ser	missense_variant	Uncertain	NO
chr1	156135970	C	T	*LMNA*	c.1006C>T	p.Arg336Trp	missense_variant	NA	NO
chr1	156135980	C	A	*LMNA*	c.1016C>A	p.Ala339Glu	missense_variant	NA	NO
chr1	156135980	C	A	*LMNA*	c.1016C>A	p.Ala339Glu	missense_variant	NA	AF and FLU
chr1	156135980	C	A	*LMNA*	c.1016C>A	p.Ala339Glu	missense_variant	NA	NO
chr1	156135992	G	A	*LMNA*	c.1028G>A	p.Arg343Gln	missense_variant	Uncertain	NO
chr1	156135992	G	A	*LMNA*	c.1028G>A	p.Arg343Gln	missense_variant	Uncertain	NO
chr1	156136044	G	C	*LMNA*	c.1080G>C	p.Gln360His	missense_variant	NA	NO
chr1	156136216	T	TACGCCTGTCCCCCAGCCC	*LMNA*	c.1161_1178dupACGCCTGTCCCCCAGCCC	p.Pro393_Thr394insArgLeuSerProSerPro	disruptive_inframe_insertion	Uncertain	NO
chr1	156136216	T	TACGCCTGTCCCCCAGCCC	*LMNA*	c.1161_1178dupACGCCTGTCCCCCAGCCC	p.Pro393_Thr394insArgLeuSerProSerPro	disruptive_inframe_insertion	Uncertain	NO
chr1	156136243	A	T	*LMNA*	c.1187A>T	p.Gln396Leu	missense_variant	Uncertain	NO
chr1	156136245	C	T	*LMNA*	c.1189C>T	p.Arg397Cys	missense_variant	Uncertain	NO
chr1	156136246	G	A	*LMNA*	c.1190G>A	p.Arg397His	missense_variant	Uncertain	NO
chr1	156136252	G	A	*LMNA*	c.1196G>A	p.Arg399His	missense_variant	Uncertain	NO
chr1	156136252	G	A	*LMNA*	c.1196G>A	p.Arg399His	missense_variant	Uncertain	NO
chr1	156136252	G	A	*LMNA*	c.1196G>A	p.Arg399His	missense_variant	Uncertain	NO
chr1	156136252	G	A	*LMNA*	c.1196G>A	p.Arg399His	missense_variant	Uncertain	NO
chr1	156136252	G	A	*LMNA*	c.1196G>A	p.Arg399His	missense_variant	Uncertain	NO
chr1	156136252	G	A	*LMNA*	c.1196G>A	p.Arg399His	missense_variant	Uncertain	NO
chr1	156136252	G	A	*LMNA*	c.1196G>A	p.Arg399His	missense_variant	Uncertain	NO
chr1	156136252	G	A	*LMNA*	c.1196G>A	p.Arg399His	missense_variant	Uncertain	NO
chr1	156136252	G	A	*LMNA*	c.1196G>A	p.Arg399His	missense_variant	Uncertain	NO
chr1	156136252	G	A	*LMNA*	c.1196G>A	p.Arg399His	missense_variant	Uncertain	NO
chr1	156136252	G	A	*LMNA*	c.1196G>A	p.Arg399His	missense_variant	Uncertain	AF and FLU
chr1	156136252	G	A	*LMNA*	c.1196G>A	p.Arg399His	missense_variant	Uncertain	NO
chr1	156136252	G	A	*LMNA*	c.1196G>A	p.Arg399His	missense_variant	Uncertain	NO
chr1	156136252	G	A	*LMNA*	c.1196G>A	p.Arg399His	missense_variant	Uncertain	NO
chr1	156136252	G	A	*LMNA*	c.1196G>A	p.Arg399His	missense_variant	Uncertain	NO
chr1	156136252	G	A	*LMNA*	c.1196G>A	p.Arg399His	missense_variant	Uncertain	NO
chr1	156136252	G	A	*LMNA*	c.1196G>A	p.Arg399His	missense_variant	Uncertain	NO
chr1	156136252	G	A	*LMNA*	c.1196G>A	p.Arg399His	missense_variant	Uncertain	NO
chr1	156136252	G	A	*LMNA*	c.1196G>A	p.Arg399His	missense_variant	Uncertain	NO
chr1	156136252	G	A	*LMNA*	c.1196G>A	p.Arg399His	missense_variant	Uncertain	NO
chr1	156136257	C	T	*LMNA*	c.1201C>T	p.Arg401Cys	missense_variant	Uncertain	NO
chr1	156136257	C	T	*LMNA*	c.1201C>T	p.Arg401Cys	missense_variant	Uncertain	NO
chr1	156136284	C	G	*LMNA*	c.1228C>G	p.Gln410Glu	missense_variant	NA	NO
chr1	156136287	G	T	*LMNA*	c.1231G>T	p.Gly411Cys	missense_variant	Uncertain	NO
chr1	156136287	G	T	*LMNA*	c.1231G>T	p.Gly411Cys	missense_variant	Uncertain	NO
chr1	156136293	G	A	*LMNA*	c.1237G>A	p.Gly413Ser	missense_variant	NA	NO
chr1	156136293	G	A	*LMNA*	c.1237G>A	p.Gly413Ser	missense_variant	NA	NO
chr1	156136299	G	A	*LMNA*	c.1243G>A	p.Val415Ile	missense_variant	Uncertain	NO
chr1	156136299	G	A	*LMNA*	c.1243G>A	p.Val415Ile	missense_variant	Uncertain	NO
chr1	156136299	G	A	*LMNA*	c.1243G>A	p.Val415Ile	missense_variant	Uncertain	NO
chr1	156136311	C	T	*LMNA*	c.1255C>T	p.Arg419Cys	missense_variant	Uncertain	PT
chr1	156136312	G	A	*LMNA*	c.1256G>A	p.Arg419His	missense_variant	Uncertain	Other arrh
chr1	156136326	A	G	*LMNA*	c.1270A>G	p.Thr424Ala	missense_variant	Uncertain	AF and FLU
chr1	156136335	C	T	*LMNA*	c.1279C>T	p.Arg427Cys	missense_variant	Uncertain	NO
chr1	156136335	C	T	*LMNA*	c.1279C>T	p.Arg427Cys	missense_variant	Uncertain	AV and LBBB
chr1	156136335	C	T	*LMNA*	c.1279C>T	p.Arg427Cys	missense_variant	Uncertain	NO
chr1	156136335	C	T	*LMNA*	c.1279C>T	p.Arg427Cys	missense_variant	Uncertain	NO
chr1	156136335	C	T	*LMNA*	c.1279C>T	p.Arg427Cys	missense_variant	Uncertain	NO
chr1	156136335	C	T	*LMNA*	c.1279C>T	p.Arg427Cys	missense_variant	Uncertain	NO
chr1	156136335	C	T	*LMNA*	c.1279C>T	p.Arg427Cys	missense_variant	Uncertain	NO

chr1	156136335	C	T	*LMNA*	c.1279C>T	p.Arg427Cys	missense_variant	Uncertain	Other arrh
chr1	156136356	G	A	*LMNA*	c.1300G>A	p.Ala434Thr	missense_variant	Uncertain	NO
chr1	156136359	C	T	*LMNA*	c.1303C>T	.	structural_interaction_variant	Pathogenic	NO
chr1	156136359	C	T	*LMNA*	c.1303C>T	.	structural_interaction_variant	Pathogenic	NO
chr1	156136362	A	G	*LMNA*	c.1306A>G	p.Thr436Ala	missense_variant	Uncertain	NO
chr1	156136368	G	A	*LMNA*	c.1311+1G>A	.	splice donor & intron variant	NA	NO
chr1	156136368	G	A	*LMNA*	c.1311+1G>A	.	splice donor & intron variant	NA	NO
chr1	156136368	G	A	*LMNA*	c.1311+1G>A	.	splice donor & intron variant	NA	NO
chr1	156136368	G	A	*LMNA*	c.1311+1G>A	.	splice donor & intron variant	NA	NO
chr1	156136371	C	A	*LMNA*	c.1315C>A	.	structural_interaction_variant	NA	NO
chr1	156136371	C	A	*LMNA*	c.1315C>A	.	structural_interaction_variant	NA	NO
chr1	156136371	C	A	*LMNA*	c.1315C>A	.	structural_interaction_variant	NA	NO
chr1	156136371	C	A	*LMNA*	c.1315C>A	.	structural_interaction_variant	NA	NO
chr1	156136371	C	T	*LMNA*	c.1315C>T	.	structural_interaction_variant	Uncertain	NO
chr1	156136373	C	T	*LMNA*	c.1317C>T	.	structural_interaction_variant	Likely benign	NO
chr1	156136374	G	A	*LMNA*	c.1318G>A	p.Val440Met	missense_variant	Uncertain	NO
chr1	156136380	G	A	*LMNA*	c.1324G>A	.	structural_interaction_variant	Uncertain	NO
chr1	156136414	G	A	*LMNA*	c.1358G>A	.	structural_interaction_variant	Uncertain	NO
chr1	156136432	A	G	*LMNA*	c.1376A>G	p.Asn459Ser	missense_variant	Uncertain	NO
chr1	156136432	A	G	*LMNA*	c.1376A>G	p.Asn459Ser	missense_variant	Uncertain	NO
chr1	156136432	A	G	*LMNA*	c.1376A>G	p.Asn459Ser	missense_variant	Uncertain	NO
chr1	156136930	A	G	*LMNA*	c.1390A>G	.	structural_interaction_variant	Uncertain	NO
chr1	156136953	C	G	*LMNA*	c.1413C>G	.	structural_interaction_variant	Likely benign	Other CD
chr1	156136953	C	G	*LMNA*	c.1413C>G	.	structural_interaction_variant	Likely benign	NO
chr1	156136953	C	G	*LMNA*	c.1413C>G	.	structural_interaction_variant	Likely benign	NO
chr1	156136974	G	C	*LMNA*	c.1434G>C	p.Leu478Phe	missense_variant	NA	NO
chr1	156136984	C	T	*LMNA*	c.1444C>T	p.Arg482Trp	missense_variant	Pathogenic	AV and LBBB
chr1	156136993	C	G	*LMNA*	c.1453C>G	p.Pro485Ala	missense_variant	Uncertain	NO
chr1	156137028	G	A	*LMNA*	c.1488G>A	.	structural_interaction_variant	Uncertain	NO
chr1	156137028	G	A	*LMNA*	c.1488G>A	.	structural_interaction_variant	Uncertain	NO
chr1	156137028	G	A	*LMNA*	c.1488G>A	.	structural_interaction_variant	Uncertain	NO
chr1	156137028	G	A	*LMNA*	c.1488G>A	.	structural_interaction_variant	Uncertain	AV and LBBB
chr1	156137141	A	C	*LMNA*	c.1517A>C	p.His506Pro	missense_variant	Uncertain	NO
chr1	156137141	A	C	*LMNA*	c.1517A>C	p.His506Pro	missense_variant	Uncertain	NO
chr1	156137141	A	C	*LMNA*	c.1517A>C	p.His506Pro	missense_variant	Uncertain	NO
chr1	156137141	A	C	*LMNA*	c.1517A>C	p.His506Pro	missense_variant	Uncertain	NO
chr1	156137141	A	C	*LMNA*	c.1517A>C	p.His506Pro	missense_variant	Uncertain	NO
chr1	156137141	A	C	*LMNA*	c.1517A>C	p.His506Pro	missense_variant	Uncertain	NO
chr1	156137141	A	C	*LMNA*	c.1517A>C	p.His506Pro	missense_variant	Uncertain	NO
chr1	156137191	G	A	*LMNA*	c.1567G>A	p.Gly523Arg	missense_variant	Uncertain	NO
chr1	156137191	G	A	*LMNA*	c.1567G>A	p.Gly523Arg	missense_variant	Uncertain	NO
chr1	156137191	G	A	*LMNA*	c.1567G>A	p.Gly523Arg	missense_variant	Uncertain	NO
chr1	156137191	G	A	*LMNA*	c.1567G>A	p.Gly523Arg	missense_variant	Uncertain	AV and LBBB
chr1	156137191	G	A	*LMNA*	c.1567G>A	p.Gly523Arg	missense_variant	Uncertain	NO
chr1	156137191	G	A	*LMNA*	c.1567G>A	p.Gly523Arg	missense_variant	Uncertain	NO

chr1	156137191	G	A	*LMNA*	c.1567G>A	p.Gly523Arg	missense_variant	Uncertain	NO
chr1	156137191	G	A	*LMNA*	c.1567G>A	p.Gly523Arg	missense_variant	Uncertain	NO
chr1	156137203	C	T	*LMNA*	c.1579C>T	.	structural_interaction_variant	Pathogenic	NO
chr1	156137204	G	A	*LMNA*	c.1580G>A	.	structural_interaction_variant	Pathogenic	NO
chr1	156137207	C	T	*LMNA*	c.1583C>T	.	structural_interaction_variant	Uncertain	NO
chr1	156137228	G	A	*LMNA*	c.1604G>A	p.Gly535Glu	missense_variant	Uncertain	NO
chr1	156137231	A	C	*LMNA*	c.1607A>C	p.Glu536Ala	missense & splice site variant	NA	NO
chr1	156137659	G	A	*LMNA*	c.1614G>A	.	structural_interaction_variant	NA	NO
chr1	156137670	A	G	*LMNA*	c.1625A>G	p.Lys542Arg	missense_variant	NA	NO
chr1	156137670	A	G	*LMNA*	c.1625A>G	p.Lys542Arg	missense_variant	NA	NO
chr1	156137678	C	T	*LMNA*	c.1633C>T	.	structural_interaction_variant	Uncertain	AF and FLU
chr1	156137678	C	A	*LMNA*	c.1633C>A	.	structural_interaction_variant	NA	NO
chr1	156137684	G	A	*LMNA*	c.1639G>A	p.Val547Met	missense_variant	NA	NO
chr1	156137690	G	A	*LMNA*	c.1645G>A	p.Val549Met	missense_variant	Uncertain	NO
chr1	156137708	G	A	*LMNA*	c.1663G>A	p.Asp555Asn	missense_variant	NA	NO
chr1	156137708	G	A	*LMNA*	c.1663G>A	p.Asp555Asn	missense_variant	NA	NO
chr1	156137738	C	T	*LMNA*	c.1693C>T	p.His565Tyr	missense_variant	NA	NO
chr1	156137756	C	A	*LMNA*	c.1711C>A	p.Arg571Ser	missense_variant	Uncertain	AV and LBBB
chr1	156137756	C	A	*LMNA*	c.1711C>A	p.Arg571Ser	missense_variant	Uncertain	NO
chr1	156137756	C	T	*LMNA*	c.1711C>T	p.Arg571Cys	missense_variant	Uncertain	NO
chr1	156138518	G	A	*LMNA*	c.1729G>A	p.Ala577Thr	missense_variant	Uncertain	NO
chr1	156138534	G	T	*LMNA*	c.1745G>T	p.Arg582Leu	missense_variant	Uncertain	NO
chr1	156138537	C	T	*LMNA*	c.1748C>T	p.Ser583Leu	missense_variant	Uncertain	NO
chr1	156138537	C	T	*LMNA*	c.1748C>T	p.Ser583Leu	missense_variant	Uncertain	NO
chr1	156138537	C	T	*LMNA*	c.1748C>T	p.Ser583Leu	missense_variant	Uncertain	NO
chr1	156138539	C	T	*LMNA*	c.1750C>T	p.Arg584Cys	missense_variant	Uncertain	NO
chr1	156138539	C	T	*LMNA*	c.1750C>T	p.Arg584Cys	missense_variant	Uncertain	AF and FLU
chr1	156138540	G	A	*LMNA*	c.1751G>A	p.Arg584His	missense_variant	Uncertain	NO
chr1	156138540	G	A	*LMNA*	c.1751G>A	p.Arg584His	missense_variant	Uncertain	NO
chr1	156138545	G	A	*LMNA*	c.1756G>A	p.Val586Met	missense_variant	Uncertain	NO
chr1	156138562	C	CG	*LMNA*	c.1776dupG	p.Gln593fs	frameshift_variant	NA	NO
chr1	156138575	G	A	*LMNA*	c.1786G>A	p.Asp596Asn	missense_variant	Uncertain	NO
chr1	156138575	G	A	*LMNA*	c.1786G>A	p.Asp596Asn	missense_variant	Uncertain	NO
chr1	156138575	G	A	*LMNA*	c.1786G>A	p.Asp596Asn	missense_variant	Uncertain	NO
chr1	156138575	G	A	*LMNA*	c.1786G>A	p.Asp596Asn	missense_variant	Uncertain	NO
chr1	156138612	G	A	*LMNA*	c.1823G>A	p.Gly608Asp	missense_variant	NA	NO
chr1	156138615	G	T	*LMNA*	c.1826G>T	p.Gly609Val	missense_variant	NA	NO
chr1	156138644	A	G	*LMNA*	c.1855A>G	p.Ser619Gly	missense_variant	NA	NO
chr1	156138645	G	A	*LMNA*	c.1856G>A	p.Ser619Asn	missense_variant	NA	NO
chr1	156138651	C	T	*LMNA*	c.1862C>T	p.Thr621Met	missense_variant	Uncertain	NO
chr1	156138662	A	C	*LMNA*	c.1873A>C	p.Ser625Arg	missense_variant	Uncertain	NO
chr1	156138662	A	C	*LMNA*	c.1873A>C	p.Ser625Arg	missense_variant	Uncertain	NO
chr1	156138662	A	C	*LMNA*	c.1873A>C	p.Ser625Arg	missense_variant	Uncertain	NO
chr1	156138663	G	C	*LMNA*	c.1874G>C	p.Ser625Thr	missense_variant	Uncertain	NO
chr1	156138663	G	C	*LMNA*	c.1874G>C	p.Ser625Thr	missense_variant	Uncertain	NO
chr1	156138663	G	C	*LMNA*	c.1874G>C	p.Ser625Thr	missense_variant	Uncertain	NO
chr1	156138668	C	T	*LMNA*	c.1879C>T	p.Arg627Cys	missense_variant	Uncertain	NO
chr1	156138668	C	T	*LMNA*	c.1879C>T	p.Arg627Cys	missense_variant	Uncertain	NO
chr1	156138669	G	A	*LMNA*	c.1880G>A	p.Arg627His	missense_variant	Uncertain	NO
chr1	156138675	T	G	*LMNA*	c.1886T>G	p.Val629Gly	missense_variant	NA	NO
chr1	156138700	C	A	*LMNA*	c.1911C>A	p.Phe637Leu	missense_variant	Uncertain	NO
chr1	156138749	C	T	*LMNA*	c.1960C>T	p.Arg654*	stop_gained	Uncertain	NO
chr1	156138749	C	T	*LMNA*	c.1960C>T	p.Arg654*	stop_gained	Uncertain	NO
chr1	156138749	C	T	*LMNA*	c.1960C>T	p.Arg654*	stop_gained	Uncertain	NO
chr1	156138749	C	T	*LMNA*	c.1960C>T	p.Arg654*	stop_gained	Uncertain	NO
chr1	156138749	C	T	*LMNA*	c.1960C>T	p.Arg654*	stop_gained	Uncertain	NO
chr1	156138749	C	T	*LMNA*	c.1960C>T	p.Arg654*	stop_gained	Uncertain	NO
chr1	156139769	G	C	*LMNA*	c.1656-1G>C	.	splice acceptor & intron variant	Na	NO

chr1	156139769	G	C	*LMNA*	c.1656-1G>C	.	splice acceptor & intron variant	Na	NO
chr1	156139770	A	G	*LMNA*	c.1656A>G	p.Ile552Met	missense & splice site variant	Na	NO
chr1	156139770	A	G	*LMNA*	c.1656A>G	p.Ile552Met	missense & splice site variant	Na	NO
chr1	156139771	CAA	C	*LMNA*	c.1658_1659 delAA	p.Gln553fs	frameshift & splice site variant	Na	NO
chr1	156139771	CAA	C	*LMNA*	c.1658_1659 delAA	p.Gln553fs	frameshift & splice site variant	Na	NO
chr1	156139771	CAA	C	*LMNA*	c.1658_1659 delAA	p.Gln553fs	frameshift & splice site variant	Na	NO
chr1	156139771	CAA	C	*LMNA*	c.1658_1659 delAA	p.Gln553fs	frameshift & splice site variant	Na	NO
chr1	156139771	CAA	C	*LMNA*	c.1658_1659 delAA	p.Gln553fs	frameshift & splice site variant	Na	NO
chr1	156139771	CAA	C	*LMNA*	c.1658_1659 delAA	p.Gln553fs	frameshift & splice site variant	Na	NO
chr1	156139775	A	G	*LMNA*	c.1661A>G	p.Glu554Gly	missense_variant	Na	NO
chr1	156139778	T	C	*LMNA*	c.1664T>C	p.Met555Thr	missense_variant	Uncertain	NO
chr1	156139813	A	C	*LMNA*	c.1699A>C	p.Lys567Gln	missense_variant	Na	NO
chr1	156139813	A	C	*LMNA*	c.1699A>C	p.Lys567Gln	missense_variant	Na	NO
chr1	156139813	A	C	*LMNA*	c.1699A>C	p.Lys567Gln	missense_variant	Na	NO
chr1	156139817	T	G	*LMNA*	c.1703T>G	p.Val568Gly	missense_variant	Na	NO
chr1	156139828	T	C	*LMNA*	c.1714T>C	p.Cys572Arg	missense_variant	Uncertain	NO

Table S3.UK Biobank WES analysis

Legend: **AT**, atrial tachycardia; **Chr,** chromossome; **Ref,** reference; **AA, a**mino acid; **CDS,** coding sequence; **NA,** not applicable; **AF,** atrial fibrillation; **FLU,** flutter; **arrh,** arrhythmia; **CD,** conduction disease; **AV,** atrioventricular; **LBBB,** left bundle branch block.
